# Development and feasibility testing of action observation training videos in acute stroke survivors: Preliminary findings

**DOI:** 10.12688/f1000research.118969.2

**Published:** 2023-02-09

**Authors:** Arunima Biswas, Manikandan Natarajan, Sandeep K Subramanian, John M. Solomon

**Affiliations:** 1 Department of Physiotherapy, Manipal College of Health Professions,Manipal Academy of Higher Education, Manipal, Karnataka, 576104, India; 2 Centre for Comprehensive Stroke Rehabilitation and Research, Manipal Academy of Higher Education, Manipal, 576104, India; 3 Departments of Physical Therapy, Physician Assistant Studies and Rehabilitation Medicine, UT Health San Antonio, San Antonio, Texas, USA

**Keywords:** action observation, feasibility, lower extremity, video

## Abstract

**Background:** Action observation training (AOT) is used for lower limb (LL) stroke rehabilitation in subacute and chronic stages, but concise information regarding the types of activities to be used and the feasibility of administration in the acute stroke population is unknown. The aim of this study was to develop and validate videos of appropriate activities for LL AOT and test administrative feasibility in acute stroke.

**Method**: A video inventory of LL activities was created after a literature survey and expert scrutiny. Five stroke rehabilitation experts validated the videos per domains of relevance, comprehension, clarity, camera position and brightness. LL AOT was then tested on ten individuals with acute stroke for uncovering barriers for clinical use in a feasibility study. Participants watched the activities and attempted imitation of the same. Determination of administrative feasibility was undertaken via participant interviews.

**Results:** Suitable LL activities for stroke rehabilitation were identified. Content validation of videos led to improvements in selected activities and video quality. Expert scrutiny led to further video processing to include different perspectives of view and speeds of projected movements. Barriers identified included inability to imitate actions shown in videos and increased distractibility for some participants.

**Conclusion:** A video catalogue of LL activities was developed and validated. AOT was deemed safe and feasible for acute stroke rehabilitation and may be used in future research and clinical practice.

## Introduction

Stroke is the second largest cause of physical disability in adults worldwide. ^1^ The ability to walk independently is a significant goal for stroke patients; however, post-stroke motor deficits like loss of muscle strength, range of motion and tonal abnormalities significantly limit the stroke survivors’ walking capacity. ^2^^,^^3^ Over the years, various rehabilitation techniques have emerged for improvement of lower limb (LL) function. A focus shift has been observed towards therapies that influence neuroplasticity in the acute stage of stroke like motor imagery. ^4^^,^^5^

Action observation training (AOT) works on the neurophysiological notion that the same neural areas are activated during observation of an action performed by another individual and execution of the same action by self. ^6^^,^^7^ LL rehabilitation has resulted in improvements in ability to walk greater distances, gait velocity and activities of daily living (ADL) in subacute to chronic stages of stroke. ^8^ It is known that the potential for recovery of motor function is maximal in the initial stages post stroke. ^9^ This is brought about by a combination of spontaneous recovery post stroke and subsequent neuroplastic changes. ^10^ Priming techniques like AOT add to this restorative process through motor learning. ^7^ Transcranial Magnetic Stimulation (TMS) studies on AOT have reported its influence on enhancing cortical excitability and promoting adaptive plasticity, if the movement practiced is specific for the desired task to be learned. ^7^ This makes AOT an intervention suitable for conditions with severe physical limitations like the acute stroke. ^8^

Individuals in the acute stage of stroke additionally experience an impairment in cognitive function. ^11^ Techniques such as AOT require active comprehension and participation from its recipients. In order to inculcate adequate motor learning, the videos of activities used for training must be suited to their level of deficit. Given that the available research revolves around subacute to chronic stroke, it is imperative to test if AOT is fit for administration in the acute stroke population. Moreover, currently, there are no activity sets designated for LL AOT in acute stroke. Therefore, there is a need to compile a collection of pertinent LL activities that can be used from the acute stage of stroke and amended as the patients improve. Hence, this study aimed to develop and validate AOT videos for LL rehabilitation and test its feasibility on acute stroke survivors.

## Methods

This is a methodological study focusing on the development of AOT videos for post-stroke LL rehabilitation. This study was approved by the Institutional Ethics Committee of Kasturba Hospital, Manipal, India (IEC 437-2019) and registered under the Clinical trials Registry of India (CTRI/2019/08/020598). The process of video development and validation was carried out as follows:

### Creation of video catalogue

**Literature survey**

The existing literature on LL AOT for stroke rehabilitation was searched to understand the extent of information available on the video content used for training. Rayyan Systems Inc. software ^12^ was used to screen AOT studies on LL stroke rehabilitation obtained from the electronic databases (See Underlying data). ^13^ The authors extracted information on types of LL activities from the included studies using the ‘descriptive-analytical’ approach and created a preliminary list of activities.

**Expert scrutiny and final inventory**

The list of activities obtained from the literature was scrutinized by two experts working in the field of stroke rehabilitation (both physiotherapists with clinical experience of five and 15 years). Addition of routine exercises and modifications based on Indian contexts (mainly regarding community ambulation) were made to the inventory by the experts based on their experience and expertise. Any conflict arising after revision of the list was resolved through consultation with another expert who has over 15 years of experience. The items agreed upon by all experts were finalized for the video catalogue.

**Video recording and processing**

A Nikon D3400 camera mounted on a tripod stand was used for recording the activities. The videos depicted men and women aged between 40 to 60 years of age, having no history of neurological illness, who willingly participated in the study. Various locations (hospital wards, home environment and community spaces) were selected for video recording, and care was taken to ensure proper brightness and the least amount of background distraction. In addition, the camera was placed at different angles to get a first-person or egocentric perspective and a third-person or allocentric perspective of the movement to encourage a clear understanding of the activities under study. The videos obtained were then grouped into six domains, namely gait prerequisites, gait initiation, walking within the hospital, walking inside home, walking out of home, and walking in the community.

### Validation of the video catalogue

Sample videos (random selection of one video from each domain) were subjected to content validation by a team of five stroke rehabilitation experts (two physiotherapists, two occupational therapists and an optometrist) having clinical experience ranging from four to 20 years. The study was explained to the experts, and they were asked to scrutinize the videos based on clarity, movement comprehensibility, relevance of activity, camera angle, background distraction, brightness, and video quality. A five-point Likert scale ranging from strongly disagree to strongly agree was used for scoring each item. ^14^ The experts were also requested to provide additional comments regarding the videos wherever appropriate.

The scores for each item and comments were analyzed. Since our panel consisted of five experts, the components having 100% agreement were accepted, while the rest were modified according to their suggestions. ^15^ The modified videos were reviewed, and the finalized video catalogue was used for feasibility testing.

### Feasibility of administering AOT in acute stroke

**Study setting and participants**

A feasibility study was carried out in the neurological unit of a tertiary care hospital in Karnataka, India. Through purposive sampling, ten individuals with stroke, as confirmed by a CT or MRI, were obtained. Individuals of either gender with stroke duration less than seven days, haemodynamically stable, who had functional vision and cognition (Montreal Cognitive Assessment score ≥ 26) were included in the study. Individuals having neglect, communication disorders, or those having previous or coexisting neurological, cardiovascular, or musculoskeletal disorders were excluded from the study. The participants were given a detailed description of the study, and written informed consent was obtained.

**Procedure**

**Figure 1.**
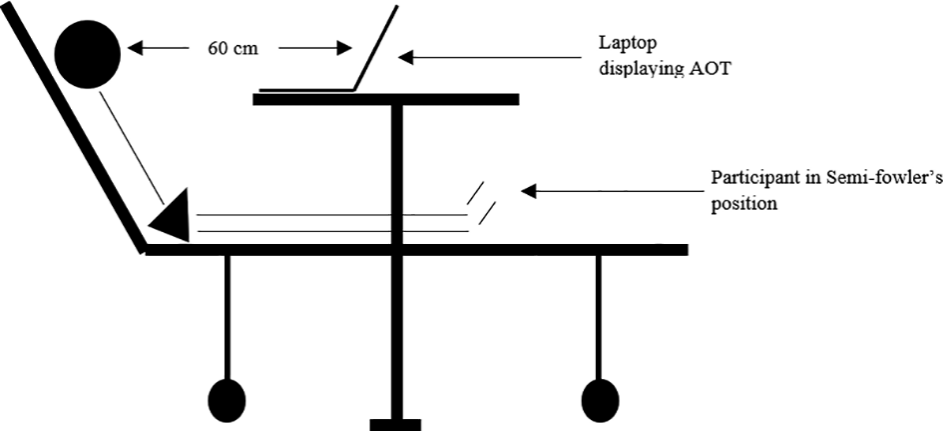
Position of the participant during action observation training.

Demographic characteristics and stroke-specific clinical parameters like LL voluntary control (VC) according to Brunnstrom grading ^16^ and Fugl Meyer Assessment for LL (FMA-LE) scores ^17^ of all participants were recorded. The AOT videos were viewed on a laptop with a 15.5-inch screen. The participants were positioned comfortably in semi fowler’s position so that the monitor was visible clearly ( Figure 1). Proper lighting and noise-free environment were ensured at all times. Participants observed side appropriate videos (corresponding to their more-affected side) on bed mobility and in-bed transitions (gait prerequisite domain). Since the participants under study belonged to the acute stroke population and did not have the capacity to walk, the AOT videos used in the study were limited to activities performed in lying or sitting position.

Ten videos of 30 seconds each were used during the intervention (See Underlying data). ^13^ The entire intervention was carried out under therapist supervision. The therapist also gave inputs to the participants regarding observation and performance of movements to ensure attention to therapy. Comprehension of observed activities was tested by asking them to identify the limb moving in the videos and explain the movement observed (either verbally or through demonstration of activity on the less-affected side.

After observing each video, participants were encouraged to imitate the movements viewed for the next 30 seconds. Participants having VC greater than two attempted five repetitions of the observed activities, while those with VC less than two performed mental practice of the movements alone. A rest period of one minute was given between observations of consecutive videos to prevent fatigue, bringing the total duration of the intervention to 20 minutes.

Post intervention, the participants were interviewed to understand their perception of the intervention using an interview guide (See Underlying data). ^13^ The feedback obtained from the interviews were compiled and analyzed. Feasibility test was done based on the following metrics: process, resource, management and scientific metrics ^18^^,^^19^ (See Underlying data). ^13^

## Results

### Development and compilation of videos

**Inventory of LL activities**

In this study, 269 unique records were obtained from the electronic search after the removal of duplicate entries. In total, 32 full-text records were screened, and nine eligible articles were included for data extraction. ^20^^–^^28^ From the included studies, 14 types of activities were identified ( Table 1).

**Table 1.** Lower extremity activities used in AOT studies.

Study	Activities
Bang et al., 2013	Walking on a treadmill
Kim & Lee, 2013	Pelvic tilting, trunk flexion & extension, trunk rotations, sit-to-stand, stand-to-sit, weight shift (front & back, left & right), lifting a foot on the block while standing, level surface gait and stepping over obstacles
Park et al., 2014	Weight shifting to the affected side, walking on straight and curved paths, walking on even and uneven surfaces, crossing obstacles, and walking with functional tasks
Park & Hwangbo, 2015	Walking on a flat land, on a slope, and on steps
Park et al., 2016	Walking on even and uneven ground, in a complex and unpredictable community environment, and in a parking lot and shopping center
Bae & Kim, 2017	Dorsiflexion of contralateral (normal) ankle
Lee et al., 2017	Knee flexion & extension, ankle dorsiflexion, hip and knee flexion with ankle dorsiflexion
Kleynen et al., 2018	Walking with different walking aids (e.g., stick, rollator)
Oh et al., 2019	Walking in a corridor, walking around the hospital, walking to the therapy room, coming out of the ward after opening and closing the door, coming in and out of toilet, shifting their blue plate, and bringing something from a refrigerator Look at different directions (front, sideways, up & down) while walking comfortably

Activities were added after expert scrutiny, mainly pertaining to in-bed mobility, balance and stepping activities that are more relevant for the acute stage of stroke before attaining walking capacity. Context-specific walking activities suited to the Indian environment were also added. Table 2 enumerates the 34 activities divided into the domains mentioned above, selected for the video catalogue.

**Table 2.** Comprehensive inventory of lower extremity activities for stroke rehabilitation.

Domain	Activities
Gait prerequisites	Supine hip-knee flexion, hip abduction, ankle dorsiflexion, bilateral & unilateral bridging, supine-to-side lying on the paretic side, supine to sit from unaffected and affected side, sitting balance, sitting knee extension, sit-to-stand, stand-to-sit & standing maintenance
Gait initiation	Standing weight shifts, forward and sideways stepping
Walking within the hospital	Cruising the sides of the hospital bed, walking around the hospital bed, walking in between 2 hospital beds in the ward
Walking inside the home	Walking from one room to another, walking to and entering a toilet, crossing thresholds within the house, walking through narrow corridors or passages; rooms with low roofs at the entrance
Walking out of home	Crossing thresholds, manoeuvring staircases and ramps, walking on different terrains like mud, gravel and concrete, crossing roads & walking in crowded areas
Walking in the community	Walking in social places (malls, hospitals, banks, temples), walking for leisure activities and in work environments

**Creation and validation of AOT videos**

A total of 130 videos, each 10 to 30 seconds long, were created based on the activities from the inventory. As mentioned above, similar videos were clubbed together in a single domain (total six domains). One video was randomly selected from each domain for content validation by experts. The level of agreement reached by the validation experts for the different scrutiny components of AOT videos is described in Table 3, along with their comments for the required modifications.

**Table 3.** Percentage Level of Agreement of items under content validity of AOT videos.

Domain	Gait prerequisite ankle toe movement	Gait initiation side stepping	Walking within the hospital, Walking in hospital ward	Walking inside the home, walking: living room to toilet	Walking out of home, crossing threshold and walking out of home (level road)	Walking in the community walking on the beach	Comments
Relevance	100	100	100	100	100	80	Avoid use of walking activities that are not part of ADLs
Movement comprehension	100	100	100	100	100	80	Use of egocentric & allocentric views of activities
Camera position	100	100	100	100	100	75	Hide models’ faces to maintain anonymity
Clarity	100	80	100	100	100	100	Focus on the moving limb
Brightness	100	80	100	100	100	100	Increase overall brightness
Video quality	100	80	100	80	100	100	
Background distraction	75	60	80	75	80	50	Use of plain white background & removal of unnecessary distractors from frame

According to experts, 100% consensus was reached regarding the relevance of activities for stroke rehabilitation except for walking in community domain. The activity deemed inappropriate (e.g., walking on the beach) was excluded from the inventory. Movement comprehension and camera position components also attained complete agreement. Domains with disagreements (clarity, background distraction, video quality and brightness) were modified according to the received suggestions. The final catalogue contained the videos processed in accordance with the expert recommendations.

### Feasibility testing of AOT

In this study, 10 respondents approached for feasibility testing of AOT participated. Table 4 describes the demographic characteristics of the participants as well as the feasibility metrics. The participant interviews carried out after the intervention revealed barriers to AOT administration in the acute stroke population including imitation inability (4/10 participants) and distractibility affecting the adherence to intervention (2/10 participants).

**Table 4.** Demographic details and feasibility metrics of participants (n = 10).

Characteristics	Value
Age in years (mean ± SD)	65.5 ± 6.4
Gender	Male: 7, Female: 3
Post stroke duration in days (mean ± SD)	3.3 ± 1.5
Paretic side	Right: 6, Left: 4
Fugl Meyer Assessment for Lower Extremity Score (mean ± SD)	11.4 ± 4.8
Voluntary Control (VC)	>2: 6, <2: 4
**Feasibility metrics**	
Process	Recruitment rate, % (n) = 100% (10/10)
Resource	Intervention duration = 25 minutes
Management	Adherence to intervention, % (n) = 80% (8/10) Adverse events = 0
Scientific	Barriers (inability to imitate), % (n) = 40% (4/10) VC > 2, % (n): 60% (6) (100% ability to imitate) VC < 2, % (n): 40% (4) (100% inability to imitate)

VC: Voluntary Control.

The hospital setting was found suitable for AOT due to the ease of video dissemination and movement practice at the bedside. All participants reported ease of comprehension of activities observed in the videos, and no adverse events were reported during the administration of the intervention.

## Discussion

In the current study, a video catalogue of various LL activities was created, and content validation was performed. This set of videos included activities at a functional task difficulty level well suited for post-stroke rehabilitation beginning at the acute stage.

### Inventory of LL activities

The existing literature on LL AOT mainly uses videos of walking in different contexts. This could be because the participants in said studies were individuals in the subacute to chronic stage of stroke with some available walking capacity. ^21^^–^^24^^,^^26^^–^^28^ Since acute stroke patients have more significant motor deficits, using mere videos of gait training may be beyond their capacity for imitation. Hence, as per suggestions from stroke rehabilitation experts, mat activities and gait prerequisite exercises were added to our inventory of LL activities to make the videos relevant for AOT through different stages of stroke.

### Development and validation of video catalogue

Although the video inventory obtained was sizeable, activities in a given domain were similar to each other, and the same parameters were used for video recording. Hence, only one video was selected from each domain randomly for content validation, as the suggested modifications were applicable to all activities of the domain. This process not only helped hasten the content validation process, but also made it less tedious for the experts.

The suggestion of the experts to use a plain white background for mat activities, keeping minimal distractors in the frame, and blurring faces of the models, is in accordance with previous studies. Simple backgrounds and removing extra objects from the frame reduce distractions and help enable better attention of the main subject. Hence, these are critical features of good quality videos. ^29^ Regarding the anonymity of models in the videos, facial blurring achieves the necessary balance between maintaining the anonymity of models and preserving the details of the activity of interest. ^30^ Specific instructions included the use of male models for activities like hip knee flexion and bridging so that proximal joints could be clearly visualized. Community ambulation videos like walking on sand were best avoided as it is not a routinely required activity in the acute stage and necessitates high-level balance function, which might not be a reality for all participants.

Furthermore, the emphasis on playing the movements at slow speed in addition to normal speed was to facilitate easier recognition of elements of complex movements and a better understanding of activities observed. ^31^ Lastly, previous studies have shown that egocentric perspective activates the hemisphere contralateral to the side of movement, while allocentric perspective activates the ipsilateral hemisphere. ^32^ Although this study did not explicitly test for this, it can be postulated that the use of egocentric and allocentric perspectives of the same movements probably facilitated the activation of the lesioned hemisphere in the form of either anatomical imitation or mirroring of activities observed.

### Feasibility of AOT in acute stroke

The results of this study suggest that the AOT can be safely administered in acute stroke and that such individuals have the capacity to understand and follow the intervention with minimal difficulty.

**Process and resource metrics**

All individuals approached for the study expressed interest in the intervention. This could be due to the use of videos as a motivating feature ^33^ or the ease of participation despite lacking significant motor function. The time required for administration of the intervention, inclusive of the observation and imitation phases, was 20 minutes. The barrier faced by four participants was inability to imitate movements. Although post-stroke impairment of imitation is mainly attributed to apraxia, ^34^^,^^35^ our participants could perform the movements with their ipsilesional leg, ruling out apraxia. Given that our participants had poor FMA scores and LL voluntary control, the difficulty faced by some participants to imitate movements might well be attributed to the same.

**Management and scientific metrics**

No falls, or other adverse events occurred during the intervention, making AOT safe for administration in acute stroke survivors in a hospital setting. Adherence to the intervention was found to be good except for distractibility in two participants. It is known that hospitalised patients in the acute stage of stroke display decreased selective attention and distractibility. ^36^ Barker-Collo *et al.* found that acute stroke patients tested poorly on various forms of attention compared to their normal counterparts. This could be the case in our participants who displayed low attention and could not adhere to the complete intervention.

To the best of our knowledge, this is one of the first studies to develop a comprehensive inventory for LL AOT and test the same on an acute stroke population. However, some limitations of this study include the lack of expert scrutiny of every video during content validation and a small sample of participants for feasibility testing. Limited generalizability of the videos having been shot in an Indian context. In addition, almost all included studies seem to be an Asian Perspective, with almost no European perspective. This, in itself, could be a limitation. Lastly, since the objective of our study was to evaluate the administrative feasibility of AOT for acute stroke, the effect of AOT on LL motor improvement and the difference in functional recovery in different types of stroke was not reported in the current study.

## Conclusion

In this study, an exhaustive video catalogue for post-stroke LL rehabilitation for different stages of stroke was created, validated, and tested for administrative feasibility in acute stroke. This system of AOT video construction may enable clinicians to formulate streamlined interventions for routine therapy. AOT was found to be safe and easy to administer in individuals with acute stroke having good cognitive capacity. Future research could focus on large scale studies testing the effect of LL AOT in acute stroke, focussing more on the recipients’ preference towards the videos used in therapy.

## Data availability

### Underlying data

Figshare: Underlying additional data. https://doi.org/10.6084/m9.figshare.19625271.v4. ^13^

This project contains the following underlying data:

Data file 1. (Literature search)

Data file 2. (Interview guide)

Data file 3. (Definition of feasibility metrics)

Data file 4. (Intervention videos)

Data are available under the CC0 1.0 Universal ( CC0 1.0) Public Domain Dedication.

## Author contributions

AB: study design and conceptualization, data collection, data analysis, manuscript writing; MN: conceptualization and study design, data analysis, manuscript writing; SKS: data analysis, manuscript writing; JMS: conceptualization and study design, data analysis and manuscript editing. All authors have read and approved the final manuscript.

## References

[1] Toell T Boehme C Mayer L : Pragmatic trial of multifaceted intervention (STROKE-CARD care) to reduce cardiovascular risk and improve quality-of-life after ischaemic stroke and transient ischaemic attack–study protocol. *BMC Neurol.* 2018;18(1):1–10. 10.1186/s12883-018-1185-2 30400876 PMC6219064

[2] Verstraeten S Mark R Sitskoorn M : Motor and cognitive impairment after stroke: a common bond or a simultaneous deficit. *Stroke Research & Therapy.* 2016;1(1):1.

[3] Hatem SM Saussez G Della Faille M : Rehabilitation of motor function after stroke: a multiple systematic review focused on techniques to stimulate upper extremity recovery. *Front. Hum. Neurosci.* 2016;10:442. 27679565 10.3389/fnhum.2016.00442 PMC5020059

[4] Belda-Lois JM Mena-del Horno S Bermejo-Bosch I : Rehabilitation of gait after stroke: a review towards a top-down approach. *J. Neuroeng. Rehabil.* 2011;8(1):1–20. 22165907 10.1186/1743-0003-8-66 PMC3261106

[5] Monteiro KB Santos CM dos Costa Cabral VR da : Effects of Motor Imagery as a Complementary Resource on the Rehabilitation of Stroke Patients: A Meta-Analysis of Randomized Trials. *J. Stroke Cerebrovasc. Dis.* 2021;30(8):105876. 10.1016/j.jstrokecerebrovasdis.2021.105876 34049014

[6] Buccino G : Action observation treatment: a novel tool in neurorehabilitation. *Philos. Trans. R. Soc. Lond., B, Biol. Sci.* 2014;369(1644):20130185. 10.1098/rstb.2013.0185 24778380 PMC4006186

[7] Stoykov ME Madhavan S : Motor priming in neurorehabilitation. *J. Neurol. Phys. Ther.* 2015;39(1):33–42. 10.1097/NPT.0000000000000065 25415551 PMC4270918

[8] Peng TH Zhu JD Chen CC : Action observation therapy for improving arm function, walking ability, and daily activity performance after stroke: a systematic review and meta-analysis. *Clin. Rehabil.* 2019;33(8):1277–1285. 10.1177/0269215519839108 30977387

[9] Zeiler SR : Should we care about early post-stroke rehabilitation? Not yet, but soon. *Curr. Neurol. Neurosci. Rep.* 2019 Mar;19(3):1–9. 10.1007/s11910-019-0927-x 30788609

[10] Felling RJ Song H : Epigenetic mechanisms of neuroplasticity and the implications for stroke recovery. *Exp. Neurol.* 2015;268:37–45. 10.1016/j.expneurol.2014.09.017 25263580 PMC4375064

[11] Grau-Olivares M Arboix A Junqué C : Progressive gray matter atrophy in lacunar patients with vascular mild cognitive impairment. *Cerebrovasc. Dis.* 2010;30(2):157–166. 10.1159/000316059 20551631

[12] Ouzzani M Hammady H Fedorowicz Z : Rayyan—a web and mobile app for systematic reviews. *Syst. Rev.* 2016;5(1):1–10. 27919275 10.1186/s13643-016-0384-4 PMC5139140

[13] Biswas A : Underlying additional data. figshare. *Media.* 2022. 10.6084/m9.figshare.19625271.v4

[14] Jebb AT Ng V Tay L : A Review of Key Likert Scale Development Advances: 1995–2019. *Front. Psychol.* 2021 [cited 2022 Apr 20];12. 10.3389/fpsyg.2021.637547 34017283 PMC8129175

[15] Polit DF Beck CT : The content validity index: are you sure you know what’s being reported? Critique and recommendations. *Res. Nurs. Health.* 2006;29(5):489–497. 10.1002/nur.20147 16977646

[16] Shah SK Harasymiw SJ Stahl PL : Stroke Rehabilitation: Outcome Based on Brunnstrom Recovery Stages. *The Occupational Therapy Journal of Research.* 1986 Nov;6(6):365–376. 10.1177/153944928600600604

[17] Hernández ED Forero SM Galeano CP : Intra- and inter-rater reliability of Fugl-Meyer Assessment of Lower Extremity early after stroke. *Braz. J. Phys. Ther.* 2021 Nov 1;25(6):709–718. 10.1016/j.bjpt.2020.12.002 33358073 PMC8721065

[18] Bowen DJ Kreuter M Spring B : How we design feasibility studies. *Am. J. Prev. Med.* 2009;36(5):452–457. 10.1016/j.amepre.2009.02.002 19362699 PMC2859314

[19] Learmonth YC Motl RW : Important considerations for feasibility studies in physical activity research involving persons with multiple sclerosis: a scoping systematic review and case study. *Pilot Feasibility Stud.* 2018;4(1):1–11. 10.1186/s40814-017-0145-8 28616252 PMC5466777

[20] Bae S Kim KY : Dual-afferent sensory input training for voluntary movement after stroke: a pilot randomized controlled study. *NeuroRehabilitation.* 2017;40(3):293–300. 10.3233/NRE-161417 28222553

[21] Kim JH Lee BH : Action observation training for functional activities after stroke: a pilot randomized controlled trial. *NeuroRehabilitation.* 2013;33(4):565–574. 10.3233/NRE-130991 24029010

[22] Park HJ Oh DW Choi JD : Action observation training of community ambulation for improving walking ability of patients with post-stroke hemiparesis: a randomized controlled pilot trial. *Clin. Rehabil.* 2017;31(8):1078–1086. 10.1177/0269215516671982 27707943

[23] Park HR Kim JM Lee MK : Clinical feasibility of action observation training for walking function of patients with post-stroke hemiparesis: a randomized controlled trial. *Clin. Rehabil.* 2014;28(8):794–803. 10.1177/0269215514523145 24569652

[24] Park EC Hwangbo G : The effects of action observation gait training on the static balance and walking ability of stroke patients. *J. Phys. Ther. Sci.* 2015;27(2):341–344. 10.1589/jpts.27.341 25729163 PMC4339133

[25] Lee HJ Kim YM Lee DK : The effects of action observation training and mirror therapy on gait and balance in stroke patients. *J. Phys. Ther. Sci.* 2017;29(3):523–526. 10.1589/jpts.29.523 28356646 PMC5361025

[26] Bang DH Shin WS Kim SY : The effects of action observational training on walking ability in chronic stroke patients: a double-blind randomized controlled trial. *Clin. Rehabil.* 2013;27(12):1118–1125. 10.1177/0269215513501528 24089434

[27] Oh SJ Lee JH Kim DH : The effects of functional action-observation training on gait function in patients with post-stroke hemiparesis: A randomized controlled trial. *Technol. Health Care.* 2019;27(2):159–165. 10.3233/THC-181388 30664512

[28] Kleynen M Jie LJ Theunissen K : The immediate influence of implicit motor learning strategies on spatiotemporal gait parameters in stroke patients: a randomized within-subjects design. *Clin. Rehabil.* 2019;33(4):619–630. 10.1177/0269215518816359 30537847

[29] Luo Y Tang X : Photo and video quality evaluation: Focusing on the subject. *European Conference on Computer Vision.* Springer;2008; p.386–399.

[30] Silas MR Grassia P Langerman A : Video recording of the operating room—is anonymity possible?. *J. Surg. Res.* 2015;197(2):272–276. 10.1016/j.jss.2015.03.097 25972314

[31] Moriuchi T Iso N Sagari A : Excitability of the primary motor cortex increases more strongly with slow-than with normal-speed presentation of actions. *PLoS One.* 2014;9(12):e114355. 10.1371/journal.pone.0114355 25479161 PMC4257605

[32] Vingerhoets G Stevens L Meesdom M : Influence of perspective on the neural correlates of motor resonance during natural action observation. *Neuropsychol. Rehabil.* 2012;22(5):752–767. 10.1080/09602011.2012.686885 22591109

[33] Cicek A Ozdincler AR Tarakci E : Interactive video game-based approaches improve mobility and mood in older adults: A nonrandomized, controlled trial. *J. Bodyw. Mov. Ther.* 2020;24(3):252–259. 10.1016/j.jbmt.2020.01.005 32825997

[34] Hoeren M Kümmerer D Bormann T : Neural bases of imitation and pantomime in acute stroke patients: distinct streams for praxis. *Brain.* 2014;137(10):2796–2810. 10.1093/brain/awu203 25062694

[35] Rumiati RI Weiss PH Tessari A : Common and differential neural mechanisms supporting imitation of meaningful and meaningless actions. *J. Cogn. Neurosci.* 2005;17(9):1420–1431. 10.1162/0898929054985374 16197695

[36] Barker-Collo SL Feigin VL Lawes CM : Attention deficits after incident stroke in the acute period: frequency across types of attention and relationships to patient characteristics and functional outcomes. *Top. Stroke Rehabil.* 2010;17(6):463–476. 10.1310/tsr1706-463 21239370

