# Supplementary material for: Development and feasibility testing of action observation training videos in acute stroke survivors: Preliminary findings
Source: F1000Res. 2023 Feb 9;11:524. Originally published 2022 May 16. [Version 2] doi: 10.12688/f1000research.118969.2 (PMC9986771; doi:10.12688/f1000research.118969.2)
Supplement: Peer review history

# Peer review: Reviewer response for version 2

**DOI:** 10.5256/f1000research.142657.r162860

**Published:** 2023-03-02

**Authors:** Sivakumar Balasubramanian

**Affiliations:** 1 Department of Bioengineering, Christian Medical College (CMC), Vellore, Tamil Nadu, India

The authors have addressed my concerns from my previous review. I have no further comments.

Is the work clearly and accurately presented and does it cite the current literature?

Yes

If applicable, is the statistical analysis and its interpretation appropriate?

Not applicable

Are all the source data underlying the results available to ensure full reproducibility?

Partly

Is the study design appropriate and is the work technically sound?

Yes

Are the conclusions drawn adequately supported by the results?

Yes

Are sufficient details of methods and analysis provided to allow replication by others?

Partly

Reviewer Expertise:

Neurorehabilitation engineering, Movement analysis

I confirm that I have read this submission and believe that I have an appropriate level of expertise to confirm that it is of an acceptable scientific standard.

# Peer review: Reviewer response for version 2

**DOI:** 10.5256/f1000research.142657.r162859

**Published:** 2023-02-09

**Authors:** Adrià Arboix

**Affiliations:** 1 Cerebrovascular Division, Department of Neurology, Hospital Universitari del Sagrat Cor, Universitat de Barcelona, Viladomat, Barcelona, Catalonia, Spain

In this revised manuscript, the authors changed it as the reviewer suggested. Now the manuscript has been significantly improved. Thank you for making these changes.

Is the work clearly and accurately presented and does it cite the current literature?

Yes

If applicable, is the statistical analysis and its interpretation appropriate?

Yes

Are all the source data underlying the results available to ensure full reproducibility?

Yes

Is the study design appropriate and is the work technically sound?

Yes

Are the conclusions drawn adequately supported by the results?

Yes

Are sufficient details of methods and analysis provided to allow replication by others?

Yes

Reviewer Expertise:

Acute stroke, cerebrovascular diseases, lacunar stroke, vascular cognitive impairment.

I confirm that I have read this submission and believe that I have an appropriate level of expertise to confirm that it is of an acceptable scientific standard.

# Peer review: Reviewer response for version 1

**DOI:** 10.5256/f1000research.130815.r154357

**Published:** 2022-12-12

**Authors:** Sivakumar Balasubramanian

**Affiliations:** 1 Department of Bioengineering, Christian Medical College (CMC), Vellore, Tamil Nadu, India

This article describes a study on the feasibility of action observation in lower-limb rehabilitation is acute stroke. The study is well-written with a simple, clean design.

 Is there some evidence for AOT in the subacute and chronic phases?

 "It is known that the potential for recovery of motor function is maximal in the acute stage of stroke." Could you provide a reference for this?

 What videos were the participants shown? Were these only the gait prerequisite videos? If not, were the patients asked to walk if they were shown videos of people walking? More details are required for the intervention. Was the intervention done under the supervision of a therapist? Did the therapists provide any comments or inputs while patients watched the video?

 When showing egocentric videos of gait, what is being shown to the participant? The view of the environment in front of them or their legs? Both of them have problems. Showing the environment might not allow them to connect it to gait. If the legs are shown, then it's unnatural. We do not look at our legs while walking. The egocentric view might make more sense for upper limb activities, but not for the lower limb.

 I have a basic question about the AOT modality. Many stroke participants who can benefit from AOT would see people walk around and do actions with their upper limbs for most of their waking hours. Why do we believe that a dedicated session of watching custom-made videos would be beneficial? This question is motivated by the reviewer's lack of a deep understanding of the AOT literature. But addressing this issue would allow other such readers to fully appreciate the rationale for the work.

 Overall, this is a good study. It can be accepted for publication after the aforementioned issues are addressed.

Is the work clearly and accurately presented and does it cite the current literature?

Yes

If applicable, is the statistical analysis and its interpretation appropriate?

Not applicable

Are all the source data underlying the results available to ensure full reproducibility?

Partly

Is the study design appropriate and is the work technically sound?

Yes

Are the conclusions drawn adequately supported by the results?

Yes

Are sufficient details of methods and analysis provided to allow replication by others?

Partly

Reviewer Expertise:

Neurorehabilitation engineering, Movement analysis

I confirm that I have read this submission and believe that I have an appropriate level of expertise to confirm that it is of an acceptable scientific standard, however I have significant reservations, as outlined above.

# Response

**Published:** 2022-12-27

**Authors:** Manikandan Natarajan

**Affiliations:** Manipal Academy of Higher Education, India

Dear reviewer,

 Thank you for taking the time to review our article and give your comments to improve the paper. The modifications done to the manuscript according to your comments are mentioned below:

**Comment 1:**

 Is there some evidence for AOT in the subacute and chronic phases?

**Response:**

 There already exists evidence for the efficacy of AOT in subacute and chronic stroke. The reviewer may peruse through the works of Tzu-Hsuan Peng *et al *(Action observation therapy for improving arm function, walking ability, and daily activity performance after stroke: a systematic review and meta-analysis), and Deirdre Ryan *et al* (Effect of Action Observation Therapy in the Rehabilitation of Neurologic and Musculoskeletal Conditions: A Systematic Review) for further clarification on the subject.

**Comment 2:**

 "It is known that the potential for recovery of motor function is maximal in the acute stage of stroke." Could you provide a reference for this?

**Response:**

 Thank you for point out this. The required reference in the introduction has been updated.

**Comment 3:**

 What videos were the participants shown? Were these only the gait prerequisite videos? If not, were the patients asked to walk if they were shown videos of people walking? More details are required for the intervention. Was the intervention done under the supervision of a therapist? Did the therapists provide any comments or inputs while patients watched the video?

**Response:**

 Thank you for the comment. The participants were shown only gait prerequisite videos (lower limb movements in sitting and lying position). The intervention was undertaken under the supervision of the treating physiotherapist and comments regarding the movements watched were also given during the therapy. The details have been added in the methods section of the article.

**Comment 4:**

 When showing egocentric videos of gait, what is being shown to the participant? The view of the environment in front of them or their legs? Both of them have problems. Showing the environment might not allow them to connect it to gait. If the legs are shown, then it's unnatural. We do not look at our legs while walking. The egocentric view might make more sense for upper limb activities, but not for the lower limb.

**Response:**

 Thank you very much for the comment and we totally agree for the same. Regarding the use of egocentric perspective of view, the participants were shown videos lower limb movements to be performed in lying or sitting positions only (as they had not yet achieved ambulation capacity). Hence the egocentric videos showed only the legs/ lower body of the model such that the participant visualizes performing the movement himself. We agree that egocentric view is more beneficial for upper limb training, but in this instance, we believed that getting an anatomically congruent view of the movement to be performed would be beneficial for the participants, as opposed to just the mirror image.

**Comment 5:**

 I have a basic question about the AOT modality. Many stroke participants who can benefit from AOT would see people walk around and do actions with their upper limbs for most of their waking hours. Why do we believe that a dedicated session of watching custom-made videos would be beneficial? This question is motivated by the reviewer's lack of a deep understanding of the AOT literature. But addressing this issue would allow other such readers to fully appreciate the rationale for the work.

**Response:**

 The success of AOT relies on the activation of mirror neuron system and mental practice by the participant. We agree that as the patients see biological movements occurring in their environment, the MNS must get activated on its own, to certain extent. However, unless dedicated observation and mental practice of the desired activities is performed (as is true with learning any new skill), the patients will not attain the required level of functioning.

 Regards & thanks,

 Dr. Manikandan Natarajan

# Peer review: Reviewer response for version 1

**DOI:** 10.5256/f1000research.130815.r148949

**Published:** 2022-09-01

**Authors:** Adrià Arboix

**Affiliations:** 1 Cerebrovascular Division, Department of Neurology, Hospital Universitari del Sagrat Cor, Universitat de Barcelona, Viladomat, Barcelona, Catalonia, Spain

The authors present a clinical study aimed at developing and validating Action Observation Training (AOT) videos for lower limb (LL) rehabilitation and testing their feasibility in acute stroke survivors. Ten patients participated in this study. A video catalog of various LL activities was created and content validation was performed. The results of this study suggest that AOT can be safely administered in acute stroke and that these individuals have the ability to understand and follow the intervention with minimal difficulty.

 This novel information is interesting from a clinical point of view. However, some amendments are suggested to improve the quality of the manuscript:

1. Due to the small sample size of the study the title should clearly mention “preliminary findings”.
2. It would be helpful to mention in the Introduction that post-stroke "cognitive impairment" is another clinical manifestation of patients with acute ischemic and hemorrhagic stroke (see and add this reference: Grau-Olivare *et al. *2010 ^1^).
3. It would be interesting to know if the authors found differences in the results obtained in patients with LL weakness secondary to pure motor hemiparesis, the most common lacunar syndrome usually due to a small subcortical infarct versus LL weakness secondary to extensive non-lacunar cerebral infarction or cerebral hemorrhage. Lacunar infarcts usually have a better spontaneous functional prognosis than other strokes (see and add this recent reference: Rudilosso *et al*. 2022 ^2^). 

Is the work clearly and accurately presented and does it cite the current literature?

Yes

If applicable, is the statistical analysis and its interpretation appropriate?

Yes

Are all the source data underlying the results available to ensure full reproducibility?

Yes

Is the study design appropriate and is the work technically sound?

Yes

Are the conclusions drawn adequately supported by the results?

Yes

Are sufficient details of methods and analysis provided to allow replication by others?

Yes

Reviewer Expertise:

Acute stroke, cerebrovascular diseases, lacunar stroke, vascular cognitive impairment.

I confirm that I have read this submission and believe that I have an appropriate level of expertise to confirm that it is of an acceptable scientific standard, however I have significant reservations, as outlined above.

## References

[1] : Progressive gray matter atrophy in lacunar patients with vascular mild cognitive impairment. *Cerebrovasc Dis* .2010;30(2) : 10.1159/000316059 157-66 10.1159/000316059 20551631

[2] : The Potential Impact of Neuroimaging and Translational Research on the Clinical Management of Lacunar Stroke. *Int J Mol Sci* .2022;23(3) : 10.3390/ijms23031497 10.3390/ijms23031497 35163423PMC8835925

# Response

**Published:** 2022-12-27

**Authors:** Manikandan Natarajan

**Affiliations:** Manipal Academy of Higher Education, India

Dear reviewer,

 Thank you for taking the time to review our article and give your comments to improve the paper. The modifications done to the manuscript according to your comments are mentioned below:

**Comment 1:**

 Due to the small sample size of the study the title should clearly mention “preliminary findings”.

**Response:**

 Thank you very much for the suggestion. We have added “preliminary findings” to our study title.

**Comment 2:**

 It would be helpful to mention in the Introduction that post-stroke "cognitive impairment" is another clinical manifestation of patients with acute ischemic and hemorrhagic stroke (see and add this reference: Grau-Olivare *et al*. 2010).

**Response:**

 Thank you for the comment. Modification in text and addition of the required reference has been done in the manuscript.

**Comment 3:**

 It would be interesting to know if the authors found differences in the results obtained in patients with LL weakness secondary to pure motor hemiparesis, the most common lacunar syndrome usually due to a small subcortical infarct versus LL weakness secondary to extensive non-lacunar cerebral infarction or cerebral hemorrhage. Lacunar infarcts usually have a better spontaneous functional prognosis than other strokes (see and add this recent reference: Rudilosso *et al*. 2022).

**Response:**

 Thank you for the comment. The aim of our study was to simply determine the feasibility of administering action observation training for lower extremity rehabilitation in the acute stage of stroke. Since our objective was not to find out the effect of AOT on the functional recovery, it is beyond the scope of this paper. However, we have clarified this detail in the limitations section of the manuscript for better clarity.

 Regards & thanks,

 Dr. Manikandan Natarajan
